# The multidimensional inventory of religious/spiritual wellbeing in Hungarian language: psychometric properties and initial validation

**DOI:** 10.3389/fpsyg.2026.1653936

**Published:** 2026-06-19

**Authors:** Bertalan Balázs Süto, Gábor Aranyi, Júlia Gyimesi, Marina Zeldovich, Human-Friedrich Unterrainer

**Affiliations:** 1 Doctoral School of Psychology, University of Pécs, Pécs, Hungary; 2 Faculty of Psychotherapy Science, Sigmund Freud University, Vienna, Austria; 3 Institute of Education and Psychology at Szombathely, ELTE Eötvös Loránd University, Budapest, Hungary; 4 Department of Personality and Clinical Psychology, Pázmány Péter Catholic University, Budapest, Hungary; 5 Institute of Psychology, University of Innsbruck, Innsbruck, Austria; 6 Addiction Research Hub (ARH), Grüner Kreis Organisation, Vienna, Austria; 7 Department of Religious Studies, University of Vienna, Vienna, Austria; 8 Department of Psychiatry and Psychotherapeutic Medicine, Medical University of Graz, Graz, Austria

**Keywords:** Hungarian language, MI-RSWB, religiosity, spirituality, subjective wellbeing, test adaptation

## Abstract

**Background:**

Research increasingly highlights the relationship between religiosity/spirituality, mental health and wellbeing. Our study presents the psychometric properties of the Hungarian version of the multidimensional inventory of religious/spiritual wellbeing (MI-RSWB) and examines its correlations with the spiritual wellbeing scale (SWBS) and the Brief Multidimensional Measurement of Religiosity Scale (BMMRS).

**Methods:**

Data were collected from 490 Hungarian participants (347 female; aged 18 and older) through an online survey. The survey included the newly translated Hungarian version of MI-RSWB, SWBS, and BMMRS.

**Results:**

Four models were tested using confirmatory factor analysis (CFA). While the 48-item version did not achieve adequate fit indicators, the 18-item abbreviated version showed acceptable fit across the tested models. The six-factor model of the Hungarian MI-RSWB demonstrated satisfactory internal consistency, in both the 48-item (α = 0.77–0.97) and 18-item (α = 0.74–0.95) versions.

**Discussion:**

The findings indicate that the Hungarian MI-RSWB has satisfying convergent and content validity and reliability. Based on the CFA results, the 18-item version is recommended for future use and research where brevity and model fit are prioritized, while the 48-item version remains useful for broader content coverage. Further validation studies are needed to assess the Hungarian 18-item MI-RSWB in both clinical and nonclinical populations.

## 1 Introduction

The aim of this paper is to present the Hungarian adaptation of the multidimensional inventory of religious/spiritual wellbeing (MI-RSWB-H). We present the translation and the psychometric indicators obtained from the adult Hungarian sample, as well as the results related to validity and reliability. The study introduces an overview of the findings derived both from the original 48-item version of the questionnaire and the 18-item version, which was developed in recent years. Furthermore, we explore the link between religion/spirituality and wellbeing, an increasingly rising topic in psychological research (Marks, 2005; Unterrainer, 2023b; VanderWeele, 2017). We treat religiosity and spirituality as key dimensions of human existence that contribute to meaning and purpose in life (Unterrainer et al., 2012). Within the bio-psycho-social-spiritual framework, religiosity and spirituality are viewed as psychological variables that influence wellbeing and other constructs. The present study intends to contribute to the understanding of these complex interactions, providing a relevant tool for examining the interplay between spirituality, religion, and wellbeing in Hungary.

Nonetheless, the creation and adaptation of these standardized tools require careful consideration, as elements of religiosity and spirituality that are nuanced by a given culture can pose significant challenges (Richards et al., 2020), which are intended to be validated across multiple languages and cultures. The MI-RSWB shows similar psychometric properties and factor structure across different European samples. However, while strong partial cross-cultural invariance was found between Austrian and Swedish samples Knorr et al. (2023); Kazemzadeh Atoofi et al. (2019) obtained different factor structures in Iranian samples.

When placing our target demographic within this broader cross-cultural framework, the Hungarian population shows no significant deviations from average European trends in terms of religious and spiritual characteristics. According to the Pew Research Center (2018), Catholicism is the largest denomination (56%), followed by Protestantism (20%), with 21% identifying as nondenominational and 3% with other affiliations. Furthermore, the survey found that 14% consider religion very important, which is the fourth lowest in Central and Eastern Europe. Hungarian census data reflects a shift away from institutionalized religions between 2001 and 2022, although the increase in religious survey respondents (from 10.8 to 40.1%) suggests a different denominational distribution when nonaffiliated individuals are considered (Tóth, 2024). Overall, religious and spiritual commitment in Hungary places it in the middle range compared to other European nations.

Within this local context, several instruments measuring various constructs in the field of psychology of religion and spirituality are already available in Hungarian. The results of these adaptation processes correspond to international trends, with only a few exceptions. These include the Spiritual Transcendence Scale (Piedmont, 1999; Tomcsányi et al., 2011), where the results obtained from the abridged version were found to be more in line with the expected outcomes (Tomcsányi et al., 2011), the Brief Multidimensional Measurement of Religiousness/Spirituality (Fetzer, 1999; Farkas et al., 2014), adapted with satisfactory results, the Age Universal I-E Scale (Gorsuch and Venable, 1983; Kézdy et al., 2018), the Post Critical Belief Scale (Horváth-Szab, 2003), the spiritual wellbeing scale (Pikó et al., 2011), and the spiritual health and life-orientation measure (SHALOM) (Fisher et al., 2000; Tornóczky et al., 2022). Historically, a significant part of the difficulties in adapting such tools has arisen from the inherent complexity of the constructs themselves (Finke and Bader, 2017). It is within this context that the multidimensional inventory of religious/spiritual wellbeing (MI-RSWB) stands out; its theoretical framework is specifically designed to capture the complexities of religiosity and spirituality by considering both the immanent and transcendent realms of human perception (Unterrainer, 2023a).

Following the recognition of a potential positive relationship between religion, spirituality, and wellbeing, accurately identifying the factors that link these domains has become an important focus in both theory and measurement (Finke and Bader, 2017). In response to this need, several key developments have emerged (Hill, 2013; Davis et al., 2024). Earlier negative hypotheses, many of which lacked empirical support, have gradually been replaced by a more inclusive approach that investigates the complex relationships between religion, spirituality, and health (Neeleman and Persaud, 1995; Marks, 2005; Shafranske, 2022). As a result, contemporary research increasingly focuses on how religious and spiritual dimensions relate to mental health, wellbeing, and overall quality of life (Falb and Pargament, 2014), presenting numerous findings that identify certain elements, such as religious participation as protective factors (VanderWeele and Ouyang, 2025). This shift in discourse highlights both the potential positive influences of religion and spirituality on human personality and wellbeing, and the continued attention within the psychology of religion/spirituality to their possible harmful effects (Pirutinsky, 2024).

### 1.1 Religiosity and Spirituality in the context of psychological wellbeing

The concept of religious/spiritual wellbeing (RSWB) can be traced back to Ellison and Paloutzian's foundational notion of spiritual wellbeing, which is supported by extensive literature and empirical research (Paloutzian et al., 2020) and which served as its key conceptual antecedent (Unterrainer et al., 2012). Building on this foundation, the integration of the bio-psycho-social-spiritual model—an inclusive view of the human experience—has provided a vital framework for further developing the concept of RSWB. This groundwork led to the creation of the multidimensional inventory for religious/spiritual wellbeing (MI-RSWB), designed to more accurately reflect the various dimensions of religious and spiritual life (Unterrainer et al., 2010). The MI-RSWB aims to integrate religious and spiritual components within a multidimensional psychological model. For a comprehensive overview of its theoretical and empirical development, see Unterrainer (2023a).

Religious/spiritual wellbeing is “the ability to experience and integrate meaning and purpose in existence through a relationship with oneself, others, or a force greater than oneself” (Unterrainer et al., 2011, p. 117). This multidimensional concept is operationalized through the multidimensional inventory for religious/spiritual wellbeing (MI-RSWB), which captures both existential (immanent) and transcendent dimensions of meaning-making. Accordingly, the instrument's structure includes six core factors. Three factors reflect transcendent aspects: general religiosity (GR), connectedness (CO), and hope transcendent (HT); three reflect immanent aspects: hope immanent (HI), forgiveness (FO), and experience of sense and meaning (SM).

GR encompasses institutionalized religious practices and traditional beliefs. CO captures a spiritual disposition, reflecting a person's tendency to feel immersed in something greater than the self. HT refers to hope in a positive afterlife, while HI reflects hope for a better future in this life. FO assesses one's ability to forgive oneself and others, and to accept past wrongs. SM includes aspects such as genuine emotion, honesty, and gratitude, indicating how meaning is perceived in daily life (see Unterrainer, 2023b for a detailed description of the sub-scales).

Scores across the six factors provide an overall measure of religious/spiritual wellbeing (RSWB) (Unterrainer et al., 2012). The full MI-RSWB scale includes 48 items, with eight items per factor. A validated short form consisting of 18 items (3 per factor) has also been developed from the original item pool, as a response to criticism regarding the length of the 48-item version (Knorr et al., 2023). As detailed by Knorr et al. (2023), when constructing the short version, items with high cross-loadings or low factor loadings were intentionally eliminated to achieve minimal error correlations and a more robust structure. Specifically, they retained items that: “*(1) best measured the underlying construct on the basis of standardized factor loadings in a six-factor* EFA *using a weighted least squares estimator (WLS); (2) demonstrated minimal cross-loadings as evidenced by the EFA modification indices; (3) exhibited minimal error correlations with other items; (4) covered a broad content range; and (5) exhibited the highest internal consistency values.”* While this rigorous item selection and reduced item heterogeneity naturally foster a cleaner factor structure and better overall model fit, it introduces a necessary trade-off regarding content coverage. For instance, reducing the items significantly narrows the conceptual breadth of certain dimensions; Knorr et al. (2023) noted that the shortened hope transcendent (HT) subscale focuses much more narrowly on the acceptance of the limitedness of life, losing some broader theoretical aspects of the original construct. Nevertheless, the shortened version has demonstrated adequate reliability (see Knorr et al., 2023; Humer et al., 2025) and the measured constructs remain largely consistent with results obtained using the full version, maintaining the core multidimensional structure.

The multidimensional structure of the scale not only advances an integrative perspective on religious and spiritual issues but also offers a useful framework for understanding their connection to human personality, mental health, and quality of life. Relationships have been identified between RSWB and variables such as attachment (e.g., Hiebler-Ragger et al., 2016; Freund et al., 2024), Big Five personality traits (e.g., Unterrainer et al., 2012; Stefa-Missagli et al., 2014; Wenzl et al., 2021), and stress and coping (e.g., Malinovic et al., 2016). Clinical samples have also been examined (see Unterrainer, 2023a). RSWB scores generally correlate positively with Extraversion, Agreeableness, Conscientiousness, and Openness to Experience, and negatively with Neuroticism. Furthermore, RSWB factors are positively associated with indicators of mental health and negatively associated with symptoms of mental illness (Unterrainer, 2023a), e.g., depression, anxiety and psychoticism (Unterrainer, 2017).

The primary objective of the present study is to present the Hungarian adaptation of the MI-RSWB and to evaluate its psychometric properties, specifically its factorial structure, reliability, and construct validity, in a nonclinical adult sample. Given that the MI-RSWB is built upon a solid theoretical framework consisting of six distinct factors (general religiosity, connectedness, hope transcendent, hope immanent, forgiveness, and experience of sense and meaning), we employed confirmatory factor analysis (CFA) to test the replicability of this established structure in the Hungarian context. This approach aligns with recent methodological advancements in the instrument's validation (e.g., Knorr et al., 2023; Stefa-Missagli et al., 2014; Wenzl et al., 2021), where CFA is utilized to rigorously test the structural stability and replicability of the MI-RSWB across different cultural and linguistic.

While the core six factors remain consistent, the previous literature and different linguistic adaptations of the measure reported various factor solutions and scoring schemes, while both a long and a short version is in use with eight and three items per factor (with a total of 48 and 18 items), respectively. Therefore, we test a model of six correlated factors (GR, FO, HI, CO, HT, and SM) and a model of the six factors loading onto a second-order overall (RSWB-O) factor (see Unterrainer et al., 2010, 2012; Unterrainer, 2023a), which corresponds to the original structure of MI-RSWB. These are supplemented with a revised model based on Knorr et al. (2023), with GR and CO loading onto a second-order revised (RSWB-R) factor, which is correlated with FO, HI, HT, and SM first-order factors. The theoretical rationale for this structure is that the GR and CO subscales represent the “core” dimensions of transcendent perception, forming a common religious/spiritual wellbeing factor, while FO, HI, HT, and SM function as independent indicators of psychosocial or existential wellbeing without direct reference to a transcendent realm (Knorr et al., 2023)

Finally, we also investigate a structure that has been suggested for scoring in previous work (see Unterrainer et al., 2013; Unterrainer, 2023a), but has not been tested before: a model with two second-order factors, where GR, CO, and HT load onto a transcendent wellbeing dimension, and HI, FO, and SM onto an Immanent wellbeing dimension. This (Model 4) directly reflects the foundational theoretical assumption of the instrument, maintaining Ellison (1983) original differentiation of spiritual wellbeing into an immanent and a transcendent area of perception (Fuchshuber and Unterrainer, 2021; Unterrainer, 2023a). We analyze the factorial structure and reliability of each model on the full 48-item set, as well as a shortened 18-item subset that is preferred in recent use (Humer et al., 2025; Knorr et al., 2023; Straßnig et al., 2025; Wenzl et al., 2021). Evaluating these models is therefore theoretically relevant, as it provides valuable empirical support for the conceptualization of the instrument. It informs whether religious/spiritual wellbeing is more accurately operationalized as a global dimension, a framework of immanent and transcendent wellbeing, or a structure of distinct but interconnected subscales.

Drawing on the instrument's theoretical framework and prior validation studies, we formulated several a priori expectations for the Hungarian sample. Regarding the factor structure, we hypothesized that established six-factor structure would be replicable (Unterrainer, 2023a). Specifically, the MI-RSWB was systematically developed to capture the multidimensional complexity of religious and spiritual wellbeing, ultimately establishing six distinct conceptual facets as its core architecture (Unterrainer et al., 2010). While the hope transcendent (HT) factor has occasionally presented psychometric challenges in prior adaptations (see Unterrainer, 2023a), an overall robust pattern has emerged supporting the stability of these core dimensions. This functional relevance has been continuously supported by theoretically sound associations with broader psychological constructs, such as personality traits, attachment, and wellbeing. Therefore, we expected the six substantive first-order factors to emerge consistently across all tested models, regardless of which higher-order or revised model would ultimately show the best comparative fit. In terms of reliability, we expected at least acceptable internal consistency (Cronbach's α ≥ 0.70) across all subscales for both versions. Finally, to establish convergent validity, we anticipated moderate to strong positive correlations between the MI-RSWB and conceptually related measures, specifically expecting its factors to align with corresponding dimensions of the SWBS and BMMRS (e.g., General Religiosity with daily spiritual experiences or private religious practices; hope immanent with existential wellbeing).

## 2 Materials and methods

### 2.1 Translation procedure

The questionnaire was translated by two native Hungarian speakers with a high level of English proficiency. In order to verify the linguistic content, a native Hungarian speaker with expert-level Swedish language skills was also engaged to translate the questionnaire from Swedish language. The resulting translations were then compared, and a reverse translation was produced by an independent translator who was proficient in English. The English translation of the original version demonstrated a high degree of agreement with the original English version.

### 2.2 Sample and procedure

Participants were recruited via social networks, including social media platforms and university forums. All participants provided informed consent prior to taking part in the study. Demographic information was collected, including age, gender, education level, relationship status, type of residence, religion, and denomination. In addition to the newly translated MI-RSWB, participants completed two standardized questionnaires in Hungarian: the brief multidimensional measure of religiosity and spirituality (BMMRS) (Farkas et al., 2014) and the spiritual wellbeing scale (SWBS) (Pikó et al., 2011). Data collection was conducted through the Qualtrics online survey platform (qualtrics.com) and included adult participants aged 18 years and older. Participation was anonymous. The research involving human participants was reviewed and approved by the Research Ethics Committee of the Institute of Psychology at Pázmány Péter Catholic University, Hungary (Approval No. 2024_19). We analyzed data from participants who finished the questionnaire. There were no missing data.

### 2.3 Psychometric measures

The survey was designed with the goal of comprehensively examining various aspects of individual religiosity and spirituality. To this end, a shortened version of the BMMRS was included in the test material. Additionally, SWBS was incorporated as a core validation instrument alongside MI-RSWB.

#### 2.3.1 The Hungarian version of the multidimensional inventory for religious/spiritual wellbeing (MI-RSWB-H)

The original English version of the MI-RSWB (Unterrainer et al., 2012) was translated into Hungarian (see Translation Procedure). The Hungarian MI-RSWB includes the full 48-item version, following the original structure, as well as a validated short form with 18 items. Responses are given on a six-point Likert scale, ranging from 1 (totally disagree) to 6 (totally agree; see Supplementary Materials). Each of the six factors is represented by eight items in the full version and three items in the short version. The six factors can be described in more detail by means of their corresponding marker-items: *General Religiosity (“My faith gives me a feeling of security.”); Forgiveness (e.g., “There are things which I cannot forgive.” – reverse coded)*; *Hope Immanent (e.g., “I view the future with optimism.”)*; *Connectedness (e.g., “I have experienced the feeling of being absorbed into something greater.”)*; *Hope Transcendent (e.g., “I often think about the fact that I will have to leave behind my loved ones.” – reverse coded); Experience of Sense and Meaning (e.g., “I have experienced true (authentic) feelings.”)*.

The full list of items for both the original and short versions is available in English and Hungarian in the Supplement (see S1 for the original version, S2 for short version). Previous adaptations of the MI-RSWB into various languages have generally shown satisfactory to high internal consistency, with Cronbach's alpha values exceeding 0.6 for the subscales and 0.8 for the total RSWB score (Unterrainer, 2023a).

The spiritual wellbeing scale (SWBS), developed by Ellison and Paloutzian, was designed to assess an individual's spiritual wellbeing and perceived quality of life, particularly in response to growing interest in subjective wellbeing (Ellison, 1983). A shortened version was later created to improve usability (Bufford et al., 1991). The scale comprises two components: religious wellbeing, which includes five items measuring an individual's relationship with God and sense of transcendence, and existential wellbeing, which includes five items assessing a person's sense of purpose and life satisfaction. A Hungarian adaptation of the shortened version demonstrated acceptable internal consistency, with Cronbach's alpha values of 0.75 for religious wellbeing and 0.64 for existential wellbeing (Pikó et al., 2011), slightly behind the values of 0.70–0.95 indicated in the literature (Paloutzian et al., 2020) regarding other translations.

The brief multidimensional measurement of religiousness/spirituality (BMMRS) was developed by the Fetzer Institute and the U. S. National Institute on Aging to assess religious and spiritual dimensions in the context of health (Fetzer, 1999; Masters, 2020). It captures both objective aspects—such as religious affiliation and service attendance—and subjective experiences. The 11-dimensional scale includes subscales like daily spiritual experiences (e.g., “*I feel God's presence”*), meaning (e.g., “*The events in my life unfold according to a divine or greater plan”*), and values/beliefs (e.g., “*I feel a deep sense of responsibility for reducing pain and suffering in the world”*). Forgiveness, private religious practices, religious and spiritual coping, and religious support address both internal beliefs and external behaviors or supports. Additional items assess significant spiritual experiences, commitment, and overall self-ratings of religiosity and spirituality. The instrument offers a broad, multidimensional profile of an individual's religious and spiritual life. The Hungarian adaptation by Farkas et al. (2014) preserved the original factor structure and demonstrated its applicability in Hungarian-speaking population.

### 2.4 Statistical analysis

Statistical analyses were conducted in R (version 4.4.1; R Core Team, 2024), using the lavaan package (version 0.6-18; Rosseel, 2012) for confirmatory factor analysis (CFA). CFA model fit was evaluated based on established guidelines (Brown, 2015), including the Comparative Fit Index (CFI; good > 0.95, acceptable > 0.90), Tucker-Lewis Index (TLI; good > 0.95, acceptable > 0.90), Root Mean Square Error of Approximation (RMSEA; good ≤ 0.06, acceptable ≤ 0.08) with 90% confidence intervals, and the Standardized Root Mean Square Residual (SRMR; good ≤ 0.05, acceptable ≤ 0.10). CFI was interpreted as a relative fit index, representing a percent reduction in model misspecification rather than a strict cut off (van Laar and Braeken, 2021).

Discriminant validity was assessed using the Fornell–Larcker criterion, which states that the square root of a factor's average variance extracted (AVE) should exceed its correlations with other factors (Fornell and Larcker, 1981). Internal consistency was evaluated using Cronbach's alpha (acceptable: 0.70–0.95; Terwee et al., 2007) and McDonald's omega, including 95% bias-corrected and accelerated (BCa) bootstrap confidence intervals based on 1,000 samples (Dunn et al., 2014; McNeish, 2018).

CFA models were estimated using the robust maximum likelihood (MLR) estimator, with scaled chi-square statistics and robust estimates of CFI, TLI, and RMSEA (Kline, 2016). Based on previous research (Knorr et al., 2023; Unterrainer et al., 2011, 2012; Wenzl et al., 2021), we tested the following four models.

Model 1 (RSWB-6): six correlated first-order factors: general religiosity (GR), connectedness (CO), hope immanent (HI), sense and meaning (SM), forgiveness (FO), and hope transcendent (HT). Model 2 (RSWB-O): a second-order model with all six factors loading on a higher-order RSWB factor. Model 3 (RSWB-R): a revised model where GR and CO load on a second-order RSWB factor, which is correlated with HI, SM, FO, and HT. Model 4 (RSWB-TI): two second-order factors—transcendent wellbeing (TWB: GR, CO, HT) and immanent wellbeing (IWB: HI, FO, SM). Each model was estimated using both the long (48-item) and short (18-item) versions of MI-RSWB.

## 3 Results

### 3.1 Sample characteristics

Data were analyzed from a total of 490 respondents. The sample was predominantly female (70.8%), with the majority of participants aged between 18 and 24 years (62%). Most participants were university students. A large portion resided in urban areas (89.6%), nearly half were currently enrolled in higher education (45.7%), and over one-third had already attained a bachelor's degree or higher (36.1%). A majority of respondents reported being in a romantic relationship (61.4%). Regarding denominational affiliation, nearly half of the sample identified as Roman Catholic (45.1%). Approximately one-third (29.6%) reported no affiliation with any religious denomination. Other affiliations included reformed protestant (11%), evangelical (4.1%), Greek catholic (1.8%), Buddhist (1.0%), Jewish (0.4%), and other religions or denominations (6.9%). Overrepresentation of young, educated, female respondents and Catholics requires careful interpretation of the results. Detailed demographic characteristics are presented in Table 1.

**Table 1:** Sample characteristics (*N* = 490).

Variable	*n* (%)
Gender	
Female	347 (70.8)
Male	141 (28.8)
Other	2 (0.04)
Age	
18–19	106 (21.6)
20–24	198 (40.4)
25–29	31 (6.3)
30–34	15 (3.1)
35–39	8 (1.6)
40–49	61 (12.4)
50–59	53 (10.8)
60+	18 (3.8)
Level of education	
Primary	17 (3.5)
Technical	5 (1)
High school	72 (14.7)
In higher education	224 (45.7)
BA/BSc	67 (14.7)
MA/MSc	105 (21.4)
Relationship status	
Married	99 (20.2)
In a relationship	202 (41.2)
Single	164 (33.5)
Divorced	21 (4.3)
Widow	4 (0.8)
Religious affiliation	
Roman catholic	221 (45.1)
Nondenominational	145 (29.6)
Other	34 (6.9)
Protestant	54 (11)
Evangelic	20 (4.1)
Greek-catholic	9 (1.8)
Buddhist	5 (1)
Jewish	2 (0.4)

### 3.2 Confirmatory factor analysis

CFA was conducted on the full sample (*N* = 490) to evaluate the factor structure of both the 48-item and 18-item versions of the MI-RSWB across four competing models. For the 48-item version, none of the tested models met the threshold for acceptable fit based on CFI values (< 0.90) (Brown, 2015). Model 1 (RSWB-6) demonstrated poor fit, with CFI = 0.817 and TLI = 0.806. Although the RMSEA was within acceptable limits (RMSEA = 0.069; 90% CI [0.067, 0.072]), the SRMR exceeded the acceptable threshold (SRMR = 0.097). Model 2 (RSWB-O) similarly showed inadequate fit (CFI = 0.803, TLI = 0.793), despite an acceptable RMSEA (RMSEA = 0.072; 90% CI [0.069, 0.072]) and an elevated SRMR (SRMR = 0.114). Model 3 (RSWB-R) followed the same pattern, with CFI = 0.813 and TLI = 0.802, and acceptable RMSEA (RMSEA = 0.070; 90% CI [0.067, 0.073]). Model 4 (RSWB-TI) also failed to meet CFI and TLI benchmarks (CFI = 0.811, TLI = 0.801), though the RMSEA remained acceptable (RMSEA = 0.070; 90% CI [0.067, 0.073]). Complete fit indices are presented in Table 2.

**Table 2:** Fit indicators of confirmatory factor analyses of RSWB models.

Long version: 48 items (8 items per factor)						
Model	χ^2^(df)	AIC	CFI	TLI	RMSEA [90% CI]	SRMR
Null model	13,163.9 (1,128)	85,799.9	0	0	0.157 [0.155; 0.160]	0.255
Model 1: RSWB-6	3,335.5 (1,065)	74,673.8	0.817	0.806	0.069 [0.067; 0.072]	0.097
Model 2: RSWB-O^a^	3,513.2 (1,074)	74,855.0	0.803	0.793	0.072 [0.069; 0.074]	0.114
Model 3: RSWB-R^a^	3,386.8 (1,068)	74,719.3	0.813	0.802	0.070 [0.067; 0.073]	0.105
Model 4: RSWB-TI^a^	3,407.7 (1,073)	74,741.4	0.811	0.801	0.070 [0.067; 0.073]	0.109
Short version: 18 items (3 items per factor)						
Model	χ^2^(df)	AIC	CFI	TLI	RMSEA [90% CI]	SRMR
Null model	3,729.5 (153)	32,420.0	0	0	0.239 [0.233; 0.246]	0.267
Model 1: RSWB-6	344.3 (120)	28,384.3	0.942	0.926	0.065 [0.057; 0.073]	0.056
Model 2: RSWB-O	504.5 (129)	28,546.4	0.903	0.885	0.081 [0.074; 0.089]	0.104
Model 3: RSWB-R	354.8 (123)	28,390.3	0.940	0.926	0.065 [0.057; 0.073]	0.059
Model 4: RSWB-TI	418.2 (128)	28,452.3	0.925	0.910	0.072 [0.064; 0.079]	0.093

Estimator: robust maximum likelihood (MLR). For each χ^2^ test (scaled), *p* < 0.001. *N* = 490. ^a^The estimated latent variable variances include negative values (Heywood case; see Kolenikov and Bollen, 2012) – the indicated models should be interpreted with caution. Models: (1) RSWB-6 = six correlated factors: GR, general religiosity; CO, connectedness; HI, hope immanent; SM, experiences of sense and meaning; FO, forgiveness; HT, hope transcendent; (2) RSWB-O = original: six factors loading onto a single second-order factor; (3) RSWB-R = Revised: GR and CO loading onto a second-order factor correlated with HI, SM, FO, and HT; and (4) RSWB-TI: transcendent/immanent = two (correlated) second-order factors, with GR, CO, and HT loading onto the transcendent wellbeing (TWB), and HI, FO, and SM loading onto immanent wellbeing (IWB). Fit indices: AIC, akaike information criterion; CFI, comparative fit index (robust); TLI, tucker-lewis index (robust); RMSEA, root mean square error of approximation (robust); 90% CI, 90% confidence limits for RMSEA; SRMR, standardized root mean square residual.

**Figure 1:**
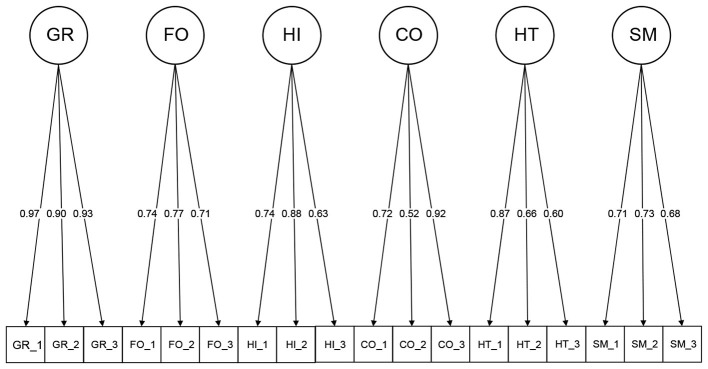
Model 1 (RSWB-6): six correlated first-order factors: GR, general religiosity; CO, connectedness; HI, hope immanent; SM, sense and meaning; FO, forgiveness; HT, hope transcendent.

**Figure 2:**
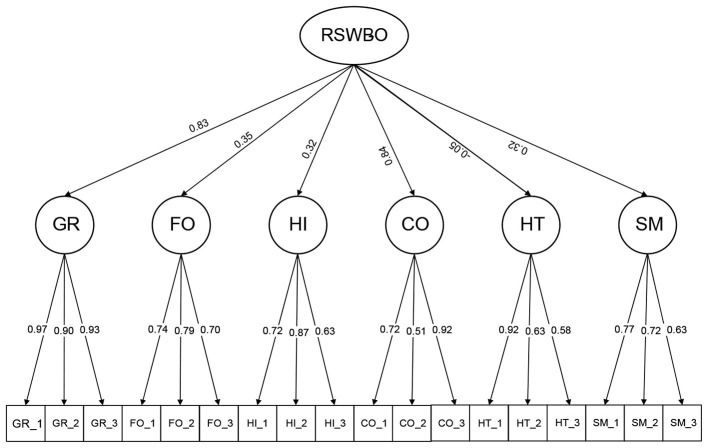
Model 2 (RSWB-O): a second-order model with all six factors loading on a higher-order RSWB factor.

**Figure 3:**
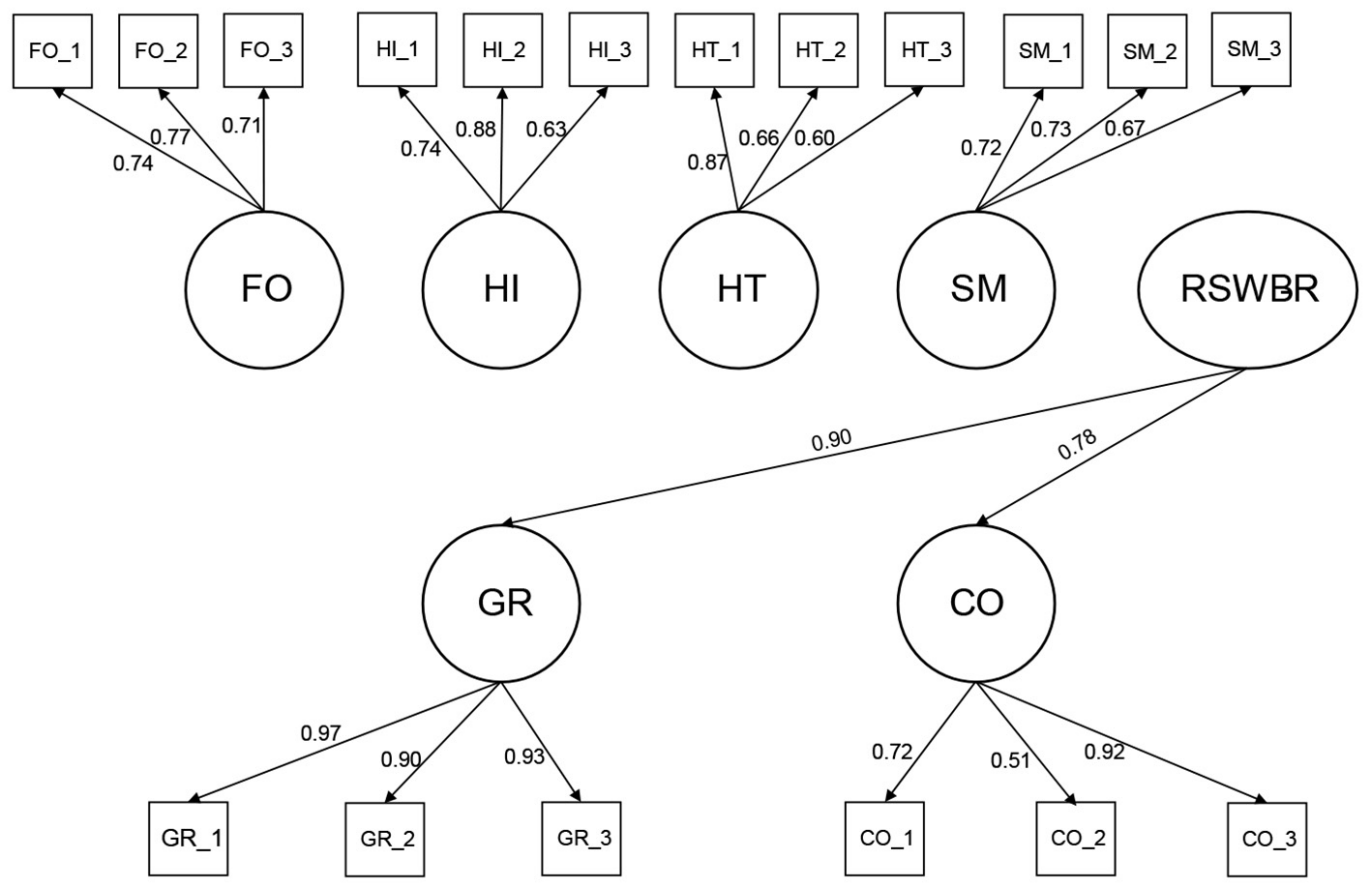
Model 3 (RSWB-R): a revised model where GR and CO load on a second-order RSWB factor, which is correlated with HI, SM, FO, and HT.

**Figure 4:**
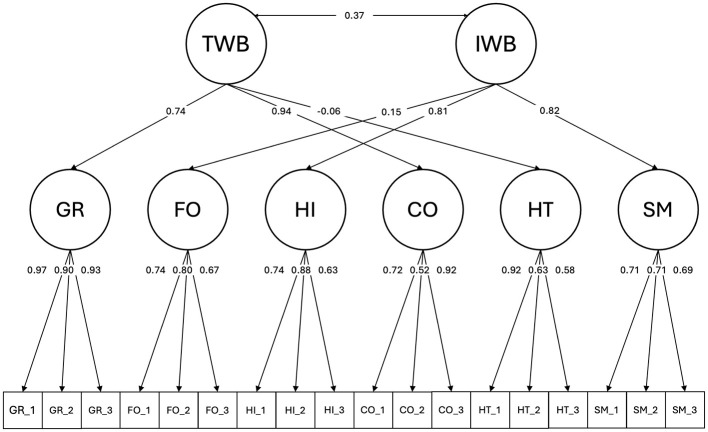
Model 4 (RSWB-TI): two second-order factors—transcendent wellbeing (TWB: GR, CO, HT) and immanent wellbeing (IWB: HI, FO, SM).

Although the 48-item version tended to show insufficient levels for most model fit indicators, the 18-item version demonstrated adequate fit across the estimated models. Model 1 (RSWB-6; see Figure 1) tested on the 18-item version showed good fit: CFI = 0.942 (acceptable), TLI = 0.926, RMSEA = 0.065 with 90% CI [0.057, 0.073], and the SRMR = 0.056. For model 2 (RSWB-O; see Figure 2) tested on the 18-item version, the indicators also suggested an acceptable model fit: CFI = 0.903 acceptable, TLI = 0.885, and both the RMSEA = 0.081 with 90% CI [0.074–0.089] and SRMR = 0.104. Model 3 (RSWB-R; see Figure 3) also exhibited satisfactory indicators, with acceptable CFI = 0.940, TLI = 0.926, and RMSEA = 0.065 with 90% CI [0.057–0.073], SRMR = 0.059. As shown in Table 3, model fit at the item level was acceptable and discriminant validity was supported. Similarly, model 4 (RSWB-TI; see Figure 4) could be considered a model with acceptable fit when tested on the 18-item version, with CFI = 0.925 (acceptable), TLI = 0.910 (acceptable), RMSEA = 0.072 with 90% CI [0.067–0.073], and SRMR = 0.093 (see Table 2).

**Table 3:** Confirmatory factor analysis of RSWB-6 (short).

Factor	Item	Mean (SD)	λ	*R* ^2^	α	AVE	Diagonal: square root of AVE. Below diagonal: latent correlations. Above diagonal: manifest correlations					
							(GR)	(FO)	(HI)	(CO)	(HT)	(SM)
General religiosity (GR)	GR_13	3.07 (1.89)	0.97	0.94	0.95	0.87	0.93	0.32^***^	0.15^**^	0.63^***^	0.00 ns	0.19^***^
	GR_19	3.18 (1.95)	0.90	0.82								
	GR_25	3.00 (1.87)	0.93	0.87								
Forgiveness (FO)	FO_02	3.76 (1.65)	0.74	0.55	0.78	0.55	0.36^***^	0.74	0.08 ns	0.19^***^	0.20^***^	0.04 ns
	FO_14	4.35 (1.70)	0.77	0.59								
	FO_20	3.77 (1.78)	0.71	0.51								
Hope immanent (HI)	HI_03	4.47 (1.24)	0.74	0.55	0.79	0.55	0.21^***^	0.12 ns	0.74	0.21^***^	−0.18^***^	0.51^***^
	HI_15	4.68 (1.10)	0.88	0.77								
	HI_27	3.94 (1.32)	0.63	0.40								
Connectedness (CO)	CO_10	3.27 (1.84)	0.72	0.51	0.75	0.57	0.70^***^	0.22^***^	0.27^***^	0.75	0.01 ns	0.22^***^
	CO_22	2.52 (1.59)	0.52	0.27								
	CO_28	3.90 (1.83)	0.92	0.85								
Hope transcendent (HT)	HT_11	2.55 (1.57)	0.87	0.76	0.75	0.52	–.02 ns	0.22^**^	–0.18^**^	−0.04 ns	0.72	–0.18^***^
	HT_17	2.34 (1.51)	0.66	0.44								
	HT_29	3.91 (1.57)	0.60	0.36								
Sense and meaning (SM)	SM_06	5.15 (1.18)	0.72	0.51	0.74	0.50	0.21^***^	0.04 ns	0.66^***^	0.29^***^	–0.28^**^	0.70
	SM_12	5.42 (1.05)	0.73	0.53								
	SM_24	4.78 (1.22)	0.68	0.46								

^*^: *p* < 0.05, ^**^: *p* < 0.01, ^***^: *p* < 0.001, ns: not statistically significant. Response format: 1–6 Likert. Item suffixes refer to each item's number on the full list (see Supplementary Files (S2) for the full list of items). λ: standardized loading. *R*^2^: proportion of variance explained in the item by the factor. α: Cronbach's alpha. AVE, average variance extracted

### 3.3 Descriptive statistics

Table 4 presents the descriptive statistics for the six RSWB factors (of the 18-item version), with the absolute values of the *z*- scores indicating statistically significant deviations from the normal distribution.

**Table 4:** Descriptive statistics of RSWB, Hungarian short version.

Factor	Women (*n* = 347)		Men (*n* = 141)		Total (*N* = 490)					
	*M*	SD	*M*	SD	*M*	SD	Min	Max	*z* skew.	*z* kurt.
GR	3.23	1.83	2.76	1.76	3.08	1.82	1	6	2.38	−6.51
FO	3.96	1.41	3.95	1.49	3.96	1.43	1	6	−3.74	−3.75
HI	4.45	0.95	4.14	1.16	4.36	1.02	1	6	−5.30	0.95
CO	3.37	1.41	2.91	1.45	3.23	1.44	1	6	0.86	−4.40
HT	2.89	1.24	3.03	1.33	2.93	1.26	1	6	4.29	−2.45
SM	5.23	0.85	4.83	1.07	5.12	0.94	1	6	−12.03	8.80
RSWB-O	3.85	0.73	3.60	0.77	3.78	0.75	2.11	5.67	2.50	−2.69
RSWB-R	3.30	1.46	2.84	1.46	3.16	1.47	1	6	1.61	−5.20
TWB	3.16	1.06	2.90	1.08	3.08	1.07	1	5.78	2.44	−3.79
IWB	4.55	0.74	4.31	0.80	4.48	0.77	2.44	6	−2.57	−2.12

GR, general religiosity; CO, connectedness; HI, hope immanent; SM, experiences of sense and meaning; FO, forgiveness; HT, hope transcendent; scale values are averages of corresponding items measured on a 1–6 Likert scale (three items per subscale). RSWB-O (original): average of all 18 items. RSWB-R (revised): average of GR and CO items. TWB, transcendental wellbeing; average of GR, CO, and HT items. IWB, immanent wellbeing; average of HI, FO, and SM items. Absolute values of skewness and kurtosis *z* scores over 1.96 indicate statistically significant departures from the normal distribution (*p* < 0.05).

### 3.4 Intercorrelations and internal consistencies

The reliability for both the 48-item (α: 0.77–0.97; ω: 0.78–0.97) and the 18-item (α: 0.74–0.95; ω: 0.75–0.95) versions was good to excellent (see Table 5), values are minimally lower for the 18-item version.

**Table 5:** Reliability coefficients of RSWB scores across models and versions.

Version (right)	Long		Short	
Factor (below)	α	ω	α	ω
General religiosity (GR)	0.97 [0.96; 0.97]	0.97 [0.96; 0.97]	0.95 [0.95; 0.96]	0.95 [0.94; 0.96]
Forgiveness (FO)	0.87 [0.85; 0.89]	0.88 [0.86; 0.89]	0.78 [0.75; 0.81]	0.78 [0.74; 0.82]
Hope immanent (HI)	0.88 [0.86; 0.90]	0.88 [0.86; 0.90]	0.79 [0.75; 0.82]	0.79 [0.74; 0.82]
Connectedness (CO)	0.78 [0.75; 0.81]	0.79 [0.75; 0.81]	0.75 [0.71; 0.79]	0.78 [0.75; 0.81]
Hope transcendent (HT)	0.77 [0.74; 0.80]	0.78 [0.74; 0.80]	0.75 [0.70; 0.78]	0.76 [0.72; 0.80]
Sense and meaning (SM)	0.82 [0.80; 0.85]	0.83 [0.79; 0.86]	0.74 [0.70; 0.78]	0.75 [0.68; 0.80]
Overall (RSWB-O)	0.91^a^ [0.90; 0.92]	0.89 [0.87; 0.91]	0.80 [0.77; 0.82]	0.79 [0.75; 0.82]
Revised (RSWB-R)	0.93 [0.92; 0.94]	0.94 [0.93; 0.94]	0.89 [0.87; 0.90]	0.89 [0.88; 0.91]
Transcendent wellbeing (TWB)	0.88^a^ [0.86; 0.90]	0.89 [0.88; 0.90]	0.79^a^ [0.76; 0.82]	0.82 [0.79; 0.84]
Immanent wellbeing (IWB)	0.87 [0.85; 0.88]	0.84 [0.80; 0.86]	0.72 [0.68; 0.75]	0.66 [0.60; 0.71]

95% confidence limits for Cronbach's alpha (α) coefficients are parametric. 95% confidence limits for McDonald's omega (ω) coefficients are bias-corrected and accelerated (BCa) based on 1000 bootstrap samples^a^: hope transcendent (HT) items negatively correlated with the scores but reversing them did improve alpha values.

Correlations among the MI-RSWB factors ranged from small to moderate, with coefficients ranging from *r* = 0.00 to *r* = 0.63 (*R*^2^ = 0.40). It is noteworthy that HT was generally uncorrelated with other factors. SM correlated moderately with HI and CO, while we observed a moderate correlation between GR and CO too. This pattern suggests that while some factors are related, others function more independently.

All factors exhibited strong correlations between the shortened 18-item version and the original 48-item version, indicating a high degree of similarity in the psychological content of the items across both versions. The RSWB total score demonstrated a strong relationship (*r* > 0.50) with the BMMRS factors of daily spiritual experiences, values/beliefs, meaning, forgiveness, private religious practices, religious and spiritual coping, overall self-rating (in terms of religiosity and spirituality), and organizational religiousness, as well as with the religious wellbeing (RWB) and existential wellbeing (EWB) factors of the SWBS, supporting global convergent validity. The GR factor also showed strong associations with these factors, except for EWB, where only a weak connection was observed (*r* = 0.21). The HI factor demonstrated a strong association exclusively with the existential factor of the SWBS (*r* = 0.72), further supporting its convergent validity. In contrast, the HT factor showed no correlation with the BMMRS or SWBS factors.

### 3.5 Gender and age effects

Pearson's correlations were calculated to explore potential age effects on the six factors. Small but statistically significant positive correlations were found between age and GR (*r* = 0.19) and FO (*r* = 0.17), while a small but statistically significant negative correlation was observed between age and SM (*r* = −0.12). Additionally, independent t-tests revealed significant differences between men and women in the following RSWB factors: general Religiosity, *t*(486) = 2.71, *p* = 0.007, *d* = 0.27; Hope Immanent, *t*(486) = 2.80, *p* = 0.005, *d* = 0.28; Connectedness, *t*(486) = 2.97, *p* = 0.003, *d* = 0.30); and sense of meaning, *t*(486) = 4.13, *p* < 0.001, *d* = 0.41).

## 4 Discussion

The primary aim of this study was to make the Hungarian version of the MI-RSWB available, along with the publication of its psychometric indicators. Based on the results, we conclude that the findings from the Hungarian sample align with trends observed in the adaptation of the MI-RSWB to other languages. The internal consistency of MI-RSWB-H factors were excellent, consistent with the results of previous adaptations summarized by Unterrainer (2023a). Small to moderate correlations between the factors support earlier findings, reinforcing the notion that the scales can be combined into a total RSWB score. Additionally, the strong correlation between the GR and CO factors, as well as the lack of correlation for the HT factor, mirror patterns reported in prior research (see Unterrainer, 2023a).

The observations of age-related correlations align with the findings of Unterrainer (2023a), including the notion that the GR and FO values may increase slightly with age.

In the light of these findings, the present study does not reveal any significant psychometric deviations specific to the Hungarian sample. Instead, the structural properties of the inventory, such as the superior model fit of the 18-item version, the distinct isolation of the HT factor, and the intercorrelation between GR and CO, closely replicates recent findings from Austrian and Swedish samples (Knorr et al., 2023; Wenzl et al., 2021). As noted in the introduction, the religious and spiritual characteristics of the Hungarian population do not deviate significantly from average European trends, which likely explains the absence of significant cultural divergence in our results (unlike adaptations in markedly different cultural contexts, e.g., Kazemzadeh Atoofi et al., 2019). Therefore, these results should be interpreted as strong evidence for the cross-cultural robustness and validity of the MI-RSWB construct across European populations. Various aspects of religiosity and spirituality were made measurable through the BMMRS as part of the test material, while the SWBS measured individuals' perceived spiritual wellbeing, however, less aspects are grasped of the construct.

The correlation between BMMRS factors and the MI-RSWB total score is a key indicator of the convergent validity of the MI-RSWB due to the degree of overlap in content between the constructs. Therefore, correlations with the factors of the BMMRS and SWBS further support the validity of the Hungarian version of MI-RSWB.

Consistent with Knorr et al. (2023), the superior model fit of the 18-item version in our sample can be attributed to the rigorous item selection process used during its development. By eliminating items with elevated cross-loadings and high error correlations, the resulting lower item heterogeneity mathematically facilitates a cleaner, more robust structure in CFA. However, reducing the item pool inevitably narrows the conceptual breadth of the constructs, such as focusing the Hope Transcendent dimension more heavily on the acceptance of life's finiteness (Knorr et al., 2023). Despite this conceptual narrowing, the strong correlations between both versions confirm that the 18-item MI-RSWB remains an adequate and structurally sound representation of the original multidimensional constructs.

Our CFA results suggest that the 18-item version is highly suitable for future applications. The development of this shortened version (Knorr et al., 2023) was based on previous findings—such as the Swedish adaptation (Wenzl et al., 2021)—which demonstrated the inadequate global fit of the full 48-item instrument. Reflecting its enhanced psychometric properties, the 18-item version is frequently used in recent research (e.g., Humer et al., 2025; Straßnig et al., 2025). However, the strict exclusion of cross-loadings during the development of the 18-item version (Knorr et al., 2023) indicates that the variable performance of the global CFA fit across different language versions and validation studies stems from the 48-item version's high structural complexity and broader conceptual breadth rather than construct invalidity. This is further supported by the fact that the full version has consistently demonstrated excellent internal consistency across diverse settings, including healthy adult populations, college students, and clinical cohorts presenting with mood or substance use disorders (Kämmerle et al., 2014; Unterrainer et al., 2011, 2013, 2014). Consequently, while the 18-item short form is preferable in contexts where brevity and model fit are prioritized, the original 48-item version remains a valuable instrument when comprehensive content coverage is important. Additionally, we propose a revised model for the shortened 18-item version (RSWB-R; see Figure 3), in which GR and CO load onto a second-order factor that is correlated with HI, SM, FO, and HT.

## 5 Limitations and future perspectives

The present sample is characterized by an overrepresentation of women and young participants. Consequently, it is not appropriate to consider this sample to be representative of the Hungarian population. In order to achieve greater representativeness in terms of age, gender and religious affiliation, more extensive data collection will be necessary in the future. It is noteworthy that highly educated, urban respondents were overrepresented, primarily due to targeted data collection among university students. However, it must also be noted that Knorr et al. (2023) identified strong gender invariance in the analysis of the 18-item version, thus suggesting the independence of the constructs from gender. When interpreting the results derived from the questionnaire data, it should be noted that participants completed the 48-item MI-RSWB instrument, and the 18-item version was evaluated as a subset of these responses. Further validation of the indicators proposed for the 18-item version should be conducted. The abridged completion time of this version would support the incorporation of other psychological constructs such as personality measurements, or analyzing test-retest reliability.

Our study provides insight into the cross-cultural validity of the MI-RSWB instrument through introducing the Hungarian version of MI-RSWB, accompanied by its psychometric indicators. The results from the Hungarian sample are consistent with trends observed in the adaptation of the MI-RSWB to other languages, confirming the instrument's broader applicability and consistency in different linguistic and cultural contexts. The internal consistency indicators of the MI-RSWB are excellent, aligning with the outcomes of previous adaptations summarized by Unterrainer (2023a). These findings highlight the reliability of the MI-RSWB, confirming its ability to accurately measure religious and spiritual wellbeing (RSWB) constructs across different cultures. Weak to moderate intercorrelations among the factors support earlier findings, indicating that the scales can be aggregated into a total RSWB score. Furthermore, the strong correlation between the GR and CO factors, along with the absence of correlation for the HT factor, replicates patterns identified in prior research (see Unterrainer, 2023a). Correlations with the BMMRS and SWBS factors further support the validity of the Hungarian version of the MI-RSWB. Our CFA results suggest that the 18-item version is highly appropriate for future applications. This conclusion aligns with previous findings summarized by Knorr et al. (2023). The recommendation to use the 18-item version of the MI-RSWB aligns with the trend in psychological research to develop shorter, more concise measurement tools, thereby facilitating broader applicability. Although the reduced number of items may have a slightly negative effect on reliability—as observed in the present dataset—internal consistency remained satisfactory across the subscales of the shortened version. Consequently, the present study recommends the Hungarian version of the 18-item MI-RSWB for future research prioritizing measurement economy, while acknowledging the continued value of the 48-item version when comprehensive conceptual depth is required. For this shortened version, we also propose a revised model (RSWB-R; see Figure 3), where GR and CO load onto a second-order factor that is correlated with HI, SM, FO, and HT. However, it is equally important to note that when examining fundamental correlations, it is beneficial to use the simpler, six-factor solution, from which a total score can be calculated (See Figure 2).

Earlier findings (see Unterrainer et al., 2012; Unterrainer, 2023a) indicate that the MI-RSWB can be applied to explore correlations between religious-spiritual wellbeing and psychological conditions (such as depression, anxiety or psychoticism), which could be extended to nonclinical samples in future research. Another notable possibility is extending in specially selected samples, such as psychotherapists (See Humer et al., 2025). In addition, further research is recommended to test measurement invariance across gender categories and age groups in a Hungarian sample, which would be best achieved through targeted data collection.

MI-RSWB, which has been validated and made available in Hungarian, contributes to research in the field of psychology of religion, supporting the exploration of the relationships between religious/spiritual wellbeing and psychological functioning. We suggest incorporating additional psychological constructs such as personality measurements in future research. Given the inherent complexity of these associations, it is recommended that these constructs be examined through the application of statistical models that are capable of uncovering a variety of indirect effects and interactions.

## Data Availability

The raw data supporting the conclusions of this article will be made available by the authors, without undue reservation.
