# Supplementary material for: The multidimensional inventory of religious/spiritual wellbeing in Hungarian language: psychometric properties and initial validation
Source: Front Psychol. 2026 Jun 19;17:1653936. doi: 10.3389/fpsyg.2026.1653936 (PMC13328269; doi:10.3389/fpsyg.2026.1653936)
Supplement: Supplementary file 1 [file Supplementary_File_1.docx]

**Supplementary material (S1)**

**The Hungarian version of MI-RSWB (MI-RSWB-H)**

**Instruction**

**Kérjük, a következő kérdések megválaszolása során tartsa szem előtt a következőket:**
1. Számos kérdést fog találni az Ön vallási/spirituális meggyőződésére vonatkozóan, és gyakran fog találkozni az „Isten” kifejezéssel.

2. Válaszait kizárólag kutatási célokra használjuk fel, Ön teljesen anonim marad.

3. Ezt a kutatást nem szponzorálta semmilyen vallási csoport, és az adatokat (sem részben, sem egészben) nem adjuk át ilyen szervezeteknek.

4. Ha kényelmetlenül érzi magát az „Isten” kifejezéssel kapcsolatban, nyugodtan helyettesítheti azt egy Önnek megfelelő kifejezéssel, mint például a „felsőbb erő”.

5. A kérdőívet akkor is kitöltheti, ha Ön agnosztikus vagy ateista nézeteket vall - a kérdőív figyelembe veszi ezeket a meggyőződéseket is. Kérjük, válaszoljon gyorsan, és próbáljon meg nem túl sokat gondolkodni egy-egy kérdésen.

Fontos továbbá, hogy minden egyes kérdésre válaszoljon; ha nem válaszol minden kérdésre, akkor a kérdőívet nem lehet megfelelően kiértékelni.

| Nr. | Dim. | Item | Nr. in short form |
| --- | --- | --- | --- |
| 1 | GR | A hitem biztonságérzetet ad nekem. |  |
| 7 | GR | Lehetséges számomra, hogy az Istennel folytatott meghitt beszélgetésekben megnyugvást találjak. |  |
| 13 | GR | Isten segítségével képes leszek felülkerekedni minden problémán. | 1 |
| 19 | GR | Az életem bizonyos pillanataiban rendkívül közel érzem magam Istenhez. | 7 |
| 25 | GR | Isten segítségével újra boldog leszek. | 13 |
| 31 | GR | Tudom, hogy Isten irgalmas. |  |
| 37 | GR | Szívesen veszek részt vallási közösségi eseményeken. |  |
| 43 | GR | Érzem Isten jelenlétét a természetben. |  |
| 2 | FO | Vannak dolgok, amiket nem tudok megbocsátani. * | 2 |
| 8 | FO | Vannak emberek, akiket gyűlölök. * |  |
| 14 | FO | Vannak emberek, akiknek sohasem fogok tudni megbocsátani. * | 8 |
| 20 | FO | Vannak dolgok, amiket az embereknek nem kellene megbocsátania. * | 14 |
| 26 | FO | Ha valaki megbánt, általában megpróbálok bosszút állni. * |  |
| 32 | FO | A gondolat, hogy az ellenségeimet szenvedni látom, elégedettséggel tölt el. * |  |
| 38 | FO | Vannak emberek, akik megérdemlik, hogy rosszul bánjanak velük. * |  |
| 44 | FO | Megbocsátottam azoknak, akik megbántottak. |  |
| 3 | HI | Optimistán tekintek a jövőbe. | 3 |
| 9 | HI | Úgy gondolom, hogy a jövőben jól alakulnak majd a dolgok. |  |
| 15 | HI | Azt hiszem, az életem jó irányba halad. | 9 |
| 21 | HI | Azt hiszem, hogy a jövőben több pozitív, mint negatív élményben lesz részem. |  |
| 27 | HI | Azt hiszem, hogy a jövőben pont úgy fogom élni az életemet, ahogy azt elképzelem. | 15 |
| 33 | HI | Pontos képem van arról, hogy milyennek kellene lennie a jövőmnek. |  |
| 39 | HI | A jövőm rendkívül bizonytalannak tűnik. * |  |
| 45 | HI | Azt hiszem, hogy a jövő izgalmas kihívásokat tartogat a számomra. |  |
| 4 | CO | Átéltem az érzést, hogy valami nagyobb egészbe olvadok bele. |  |
| 10 | CO | Hiszem, hogy újjászületek a halálom után. | 4 |
| 16 | CO | Vannak emberek, akikkel természetfeletti kapcsolatot érzek. |  |
| 22 | CO | Voltak olyan élményeim, amelyek révén rájöttem, hogy semmi sem hal meg. | 10 |
| 28 | CO | Hiszek a halál utáni létben. | 16 |
| 34 | CO | Átéltem dolgokat, amiket nem lehet szavakkal kifejezni. |  |
| 40 | CO | Tapasztaltam már, hogy vannak tárgyak, amelyek különös erőt sugároznak. |  |
| 46 | CO | Úgy hiszem, hogy lesz olyan élményem a jövőben, mely nagyon kevés embernek adatik meg. |  |
| 5 | HT | Gyakran gondolok arra a tényre, hogy hátra kell majd hagynom a szeretteimet. * |  |
| 11 | HT | Bármit megtennék, hogy meghosszabbítsam a szeretteim életét. * | 5 |
| 17 | HT | Nehéz arra gondolnom, hogy egy nap a szeretteim már nem élnek tovább. * | 11 |
| 23 | HT | Félek, hogy halálom után el fognak felejteni. * |  |
| 29 | HT | Bármit megtennék, hogy meghosszabbítsam az életemet. * | 17 |
| 35 | HT | Félek attól, hogy mi fog történni velem a halálom után. * |  |
| 41 | HT | A halállal minden remény véget ér. * |  |
| 47 | HT | Félek attól, hogy halálom után felelősségre vonnak a rossz cselekedeteimért. * |  |
| 6 | SM | Megtapasztaltam valódi (igaz) érzéseket. | 6 |
| 12 | SM | Átéltem már mély szeretetet. | 12 |
| 18 | SM | Átéltem igaz barátságot. |  |
| 24 | SM | Gyakran éltem át nyitottságot és őszinteséget. | 18 |
| 30 | SM | Volt részem olyan élményekben, amiket újra és újra szeretnék átélni. |  |
| 36 | SM | Gyakran volt részem olyan élményekben, amelyek mély hatást tettek rám. |  |
| 42 | SM | Tapasztalatom szerint, lehetséges úgy belemerülnöm valamibe, hogy mindenről megfeledkezem magam körül. |  |
| 48 | SM | Volt részem egy vagy több olyan élményben, amelyek során az élet értelme világossá vált számomra. |  |

The 48-item version of the Hungarian MI-RSWB (Multidimensional Inventory of Religious/Spiritual Well-Being)

Evaluation scheme

| Dimension | Item number |
| --- | --- |
| General Religiosity (GR) | 1,7,13,19,25,31,37,43 |
| Forgiveness (FO) | 2*,8*,14*,20*,26*,32*,38*,44 |
| Hope Immanent (HI) | 3,9,15,21,27,33,39*,45 |
| Connectedness (CO) | 4,10,16,22,28,34,40,46 |
| Hope Transcendent (HT) | 5*,11*,17*,23*,29*,35*,41*,47* |
| Experiences of Sense and Meaning (SM) | 6,12,18,24,30,36,42,48 |

***** marked items have reverse scoring

*For the English version of the MI-RSWB, see Unterrainer et al., 2012*

Unterrainer, H. F., Nelson, O., Collicutt, J., & Fink, A. (2012). The English version of the Multidimensional Inventory for Religious/Spiritual Well-being (MI-RSWB-E): First results from British college students. *Religions*, *3*(3), 588-599.

*For the German version of the MI-RSWB, see Unterrainer & Fink, 2013*

Unterrainer, H. F., & Fink, A. (2013). Das Multidimensionale Inventar zum religiös-spirituellen Befinden (MI-RSB). *Diagnostica*.
