# Supplementary material for: The multidimensional inventory of religious/spiritual wellbeing in Hungarian language: psychometric properties and initial validation
Source: Front Psychol. 2026 Jun 19;17:1653936. doi: 10.3389/fpsyg.2026.1653936 (PMC13328269; doi:10.3389/fpsyg.2026.1653936)
Supplement: Supplementary file 2 [file Supplementary_File_2.docx]

**Supplementary material (S2)**

**The Hungarian short version of MI-RSWB (MI-RSWB-H-18)**

**Instruction**

**Kérjük, a következő kérdések megválaszolása során tartsa szem előtt a következőket:**
1. Számos kérdést fog találni az Ön vallási/spirituális meggyőződésére vonatkozóan, és gyakran fog találkozni az „Isten” kifejezéssel.

2. Válaszait kizárólag kutatási célokra használjuk fel, Ön teljesen anonim marad.

3. Ezt a kutatást nem szponzorálta semmilyen vallási csoport, és az adatokat (sem részben, sem egészben) nem adjuk át ilyen szervezeteknek.

4. Ha kényelmetlenül érzi magát az „Isten” kifejezéssel kapcsolatban, nyugodtan helyettesítheti azt egy Önnek megfelelő kifejezéssel, mint például a „felsőbb erő”.

5. A kérdőívet akkor is kitöltheti, ha Ön agnosztikus vagy ateista nézeteket vall - a kérdőív figyelembe veszi ezeket a meggyőződéseket is. Kérjük, válaszoljon gyorsan, és próbáljon meg nem túl sokat gondolkodni egy-egy kérdésen.

Fontos továbbá, hogy minden egyes kérdésre válaszoljon; ha nem válaszol minden kérdésre, akkor a kérdőívet nem lehet megfelelően kiértékelni.

| Nr. | Dim. | Item | Nr. In 48 item version |
| --- | --- | --- | --- |
| 1 | GR | Isten segítségével képes leszek felülkerekedni minden problémán. | 13 |
| 7 | GR | Az életem bizonyos pillanataiban rendkívül közel érzem magam Istenhez. | 19 |
| 13 | GR | Isten segítségével újra boldog leszek. | 25 |
| 2 | FO | Vannak dolgok, amiket nem tudok megbocsátani. * | 2 |
| 8 | FO | Vannak emberek, akiknek sohasem fogok tudni megbocsátani. * | 14 |
| 14 | FO | Vannak dolgok, amiket az embereknek nem kellene megbocsátania. * | 20 |
| 3 | HI | Optimistán tekintek a jövőbe. | 3 |
| 9 | HI | Azt hiszem, az életem jó irányba halad. | 15 |
| 15 | HI | Azt hiszem, hogy a jövőben pont úgy fogom élni az életemet, ahogy azt elképzelem. | 27 |
| 4 | CO | Hiszem, hogy újjászületek a halálom után. | 10 |
| 10 | CO | Voltak olyan élményeim, amelyek révén rájöttem, hogy semmi sem hal meg. | 22 |
| 16 | CO | Hiszek a halál utáni létben. | 28 |
| 5 | HT | Bármit megtennék, hogy meghosszabbítsam a szeretteim életét. * | 11 |
| 11 | HT | Nehéz arra gondolnom, hogy egy nap a szeretteim már nem élnek tovább. * | 17 |
| 17 | HT | Bármit megtennék, hogy meghosszabbítsam az életemet. * | 29 |
| 6 | SM | Megtapasztaltam valódi (igaz) érzéseket. | 6 |
| 12 | SM | Átéltem már mély szeretetet. | 12 |
| 18 | SM | Gyakran éltem át nyitottságot és őszinteséget. | 24 |

The 18-item version of the Hungarian MI-RSWB (Multidimensional Inventory of Religious/Spiritual Well-Being)

Evaluation scheme

| Dimension | Item number |
| --- | --- |
| General Religiosity (GR) | 13,19,25, |
| Forgiveness (FO) | 2*,14*,20* |
| Hope Immanent (HI) | 3,15,27 |
| Connectedness (CO) | 10,22,28 |
| Hope Transcendent (HT) | 11*,17*,29* |
| Experiences of Sense and Meaning (SM) | 6,12,24 |

*marked items have reverse scoring

*For the 18-item version of the MI-RSWB (English) see Knorr et al, 2023*

Knorr, A., Podolin-Danner, N., Fuchshuber, J., Wenzl, M., Silani, G., & Unterrainer, H. F. (2023). Development and validation of the Multidimensional Inventory for Religious/Spiritual Well-Being 18 item version (MI-RSWB-18). *Personality and Individual Differences*, *209*, 112213.
